# Phosphorylation Impacts Cu(II) Binding by ATCUN Motifs

**DOI:** 10.1021/acs.inorgchem.1c00939

**Published:** 2021-06-07

**Authors:** Tomasz Frączyk

**Affiliations:** Institute of Biochemistry and Biophysics, Polish Academy of Sciences, Pawińskiego 5a, 02-106 Warsaw, Poland

## Abstract

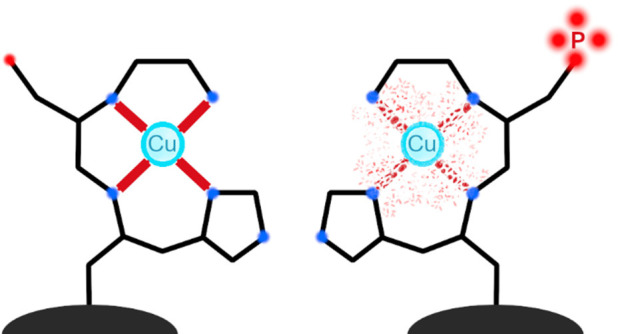

ATCUN (amino terminal Cu(II) and Ni(II) binding) motifs chelate Cu(II) ions strongly. However, the impact of the phosphorylation of neighboring residues on such complexation has not been elucidated. The copper(II) dissociation constants of original and phosphorylated peptides from human histatin-1 and human serum albumin were compared using spectroscopic methods. Phosphorylation markedly weakened Cu(II) binding. Thus, these results indicate that phosphorylation may be a vital mechanism governing metal ion binding.

## Abstract 2

The phosphorylation of serine residues within or nearby an ATCUN motif markedly weakens Cu(II) binding. It indicates that phosphorylation may be a vital mechanism of metal ion binding regulation.

More than 100 human extracellular proteins and peptides contain an ATCUN motif,^1^ which has an amino acid sequence Xaa-Zaa-His, where Xaa is any amino acid residue with a free N-terminal amine, and Zaa is any amino acid residue except proline. Human histatin-1 and human serum albumin (HSA) contain this motif–Asp-Ser-His and Asp-Ala-His, respectively. Cu(II) complexes with these molecules are biologically relevant. Such peptides bind Cu(II) strongly, with subpicomolar to femtomolar dissociation constants.^2−6^

Phosphorylation of serine residues is one of the most frequent post-translational modifications (PTMs).^7,8^ It impacts a range of protein properties, e.g., molecular dynamics, interaction with other molecules or neighboring residues, or the propensity to change the cellular compartment.^8^ However, there is no information about the impact of such phosphorylation on Cu(II) binding by the ATCUN motif.

Two hexapeptides, N-termini from human histatin-1 (DSHEKR-am) and HSA (DAHKSE-am), were chosen to test the influence of serine phosphorylation on Cu(II) binding by the ATCUN motif. Spectrophotometric methods revealed the dissociation constants for the original and modified peptides.

First, spectroscopic pH-metric titrations of Cu(II)/peptide solutions in a 0.9:1.0 ratio with UV–vis and circular dichroism (CD) spectra registration were performed (Figures 1 and 2). The appearance of *d–d* bands characteristic of 4N square planar complexes was detected by both methods. The p*K*_a_ values for the formation of 4N complexes were calculated from the pH dependence of the absorbance and CD signal (Figure 3), as reported earlier (see also the Supporting Information).^9^ Interestingly, phosphorylation caused an increase in such p*K*_a_ values by 0.3 pH units for both peptides (4.13 ± 0.04 vs 4.37 ± 0.05 for DSHEKR-am and 4.53 ± 0.04 vs 4.82 ± 0.04 for DAHKSE-am), strongly suggesting that serine phosphorylation decreases the affinity of these peptides for Cu(II).

**Figure 1:**
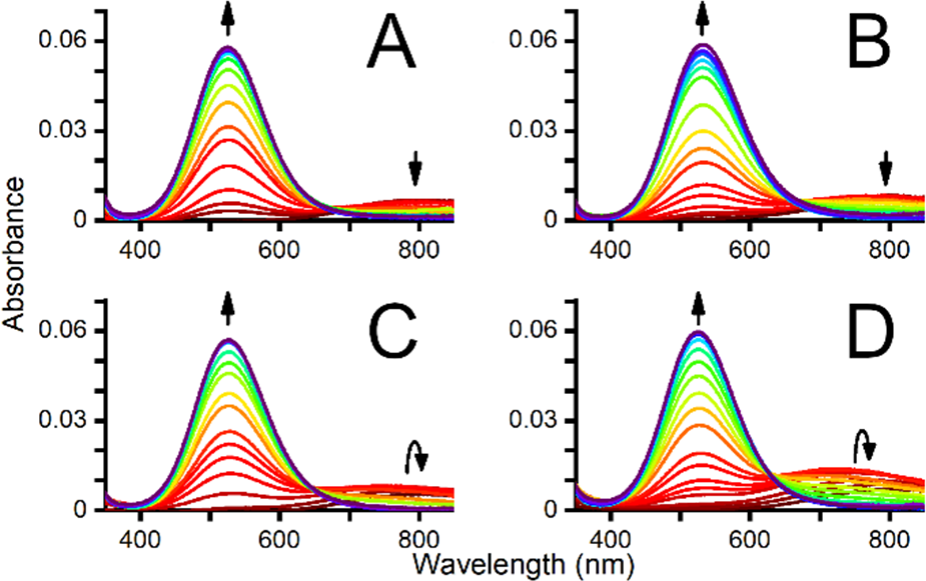
UV–vis spectra of 0.67 mM peptide and 0.60 mM CuCl_2_ in the pH range of 3 (red) to 10 (violet). The diminishing (at 800 nm) and appearing (at 530 nm) *d–d* bands with increasing pH are shown for A) DSHEKR-am, B) DpSHEKR-am, C) DAHKSE-am, and D) DAHKpSE-am.

**Figure 2:**
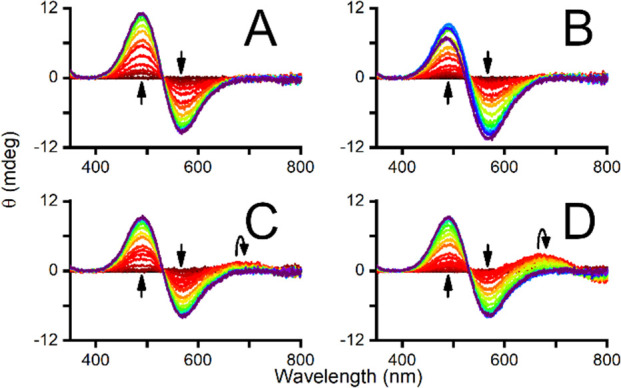
CD spectra of 0.67 mM peptide and 0.60 mM CuCl_2_ in the pH range of 3 (red) to 10 (violet). The appearance of *d–d* bands at 490 and 570 nm with increasing pH is shown for A) DSHEKR-am, B) DpSHEKR-am, C) DAHKSE-am, and D) DAHKpSE-am.

**Figure 3:**
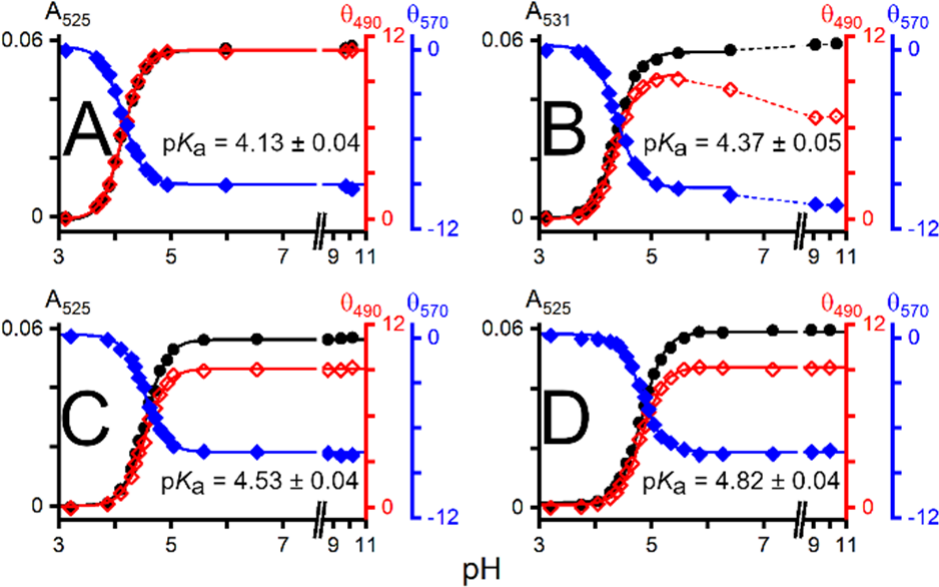
pH dependence of absorbance and circular dichroism of 0.67 mM peptide and 0.60 mM CuCl_2_ for A) DSHEKR-am, B) DpSHEKR-am, C) DAHKSE-am, and D) DAHKpSE-am. p*K*_a_ values (±SD) for forming 4N square planar complexes calculated from the data are also shown for each peptide.

According to a recently published approach,^2^ the competition of the hexapeptides and GGH tripeptide for Cu(II) ions was observed by CD spectroscopy (Figure 4) to examine the influence of serine phosphorylation on Cu(II) binding by the ATCUN motif. Briefly, CD spectra of 0.67 mM hexapeptide and 0.60 mM Cu(II) with variable concentrations of GGH at pH 7.4 (equilibrated for 24 h) were recorded. Each spectrum was decomposed to find the component concentrations: Cu(hexapeptide) and Cu(GGH) complexes allowing me to compute the relative strength of the hexapeptide with GGH as a reference (Supporting Information).

**Figure 4:**
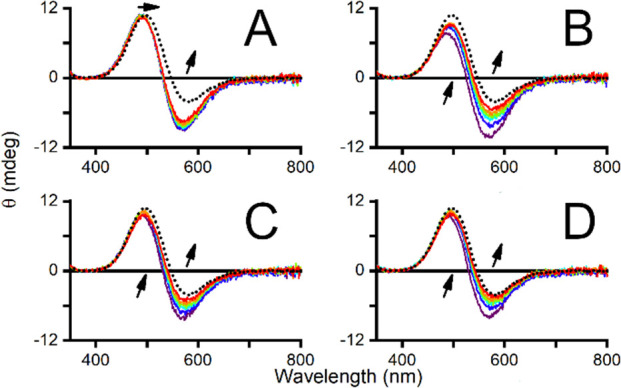
CD spectra of 0.67 mM peptide and 0.60 mM CuCl_2_ at pH 7.4 (50 mM HEPES) titrated with GGH. The redshifts of the *d–d* bands from the investigated Cu(hexapeptide) complex (violet) to Cu(GGH) (black, dotted) during the titration are shown for A) DSHEKR-am, B) DpSHEKR-am, C) DAHKSE-am, and D) DAHKpSE-am.

In detail, the linear dependence is derived from the equation
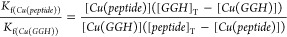
where [Cu(peptide)] and [Cu(GGH)] are measured by decomposition of experimental spectra, and [peptide]_T_ and [GGH]_T_ are the total concentrations of each ligand. The slope of this dependence is equal to the number saying how many times the formation constant (*K*_f_) for Cu(hexapeptide) is higher than for Cu(GGH) (*cf*. Figure 5 and Supporting Information). The dissociation constant of Cu(GGH) at pH 7.4 was recently determined to be 609.5 fM (log *K*_f__(*Cu*(*GGH*))_ = 12.215 ± 0.005).^2^ Thus, one can multiply *K*_f__(*Cu*(*GGH*))_ by the relevant slope and obtain the final *K*_f__(*Cu*(*hexapeptide*))_. Based on this approach, the dissociation constants are 2.2 fM (log *K*_f_ = 14.65 ± 0.12) and 38.6 fM (log *K*_f_ = 13.41 ± 0.03) for DSHEKR-am and DpSHEKR-am, respectively. Analogously, 26.9 fM (log *K*_f_ = 13.57 ± 0.05) and 60.8 fM (log *K*_f_ = 13.22 ± 0.05) dissociation constants were found for DAHKSE-am and DAHKpSE-am, respectively. Thus, phosphorylation of nearby serine residues markedly lowers the affinity of the ATCUN motif for Cu(II) ions. The effect is more pronounced for serine residues in the second position in the sequence compared with the fifth position. This seems reasonable, as serine in the second position is much closer to the coordination sphere.

**Figure 5:**
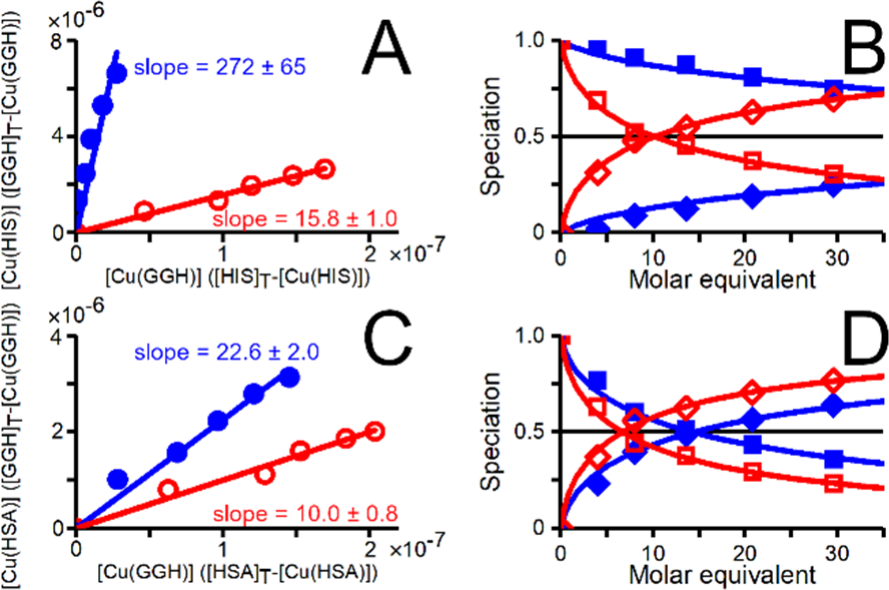
Determination of the relative Cu(II) binding strength of investigated peptides and GGH, based on the competition experiment shown in Figure 4, calculated for peptides from A) histatin-1 and C) HSA, for nonphosphorylated (blue, dots) and phosphorylated (red, circles) ones. The calculated slopes (±SD) reflect the relative Cu(II) binding affinities. Speciation diagrams were obtained by decomposing the CD spectra from Figure 4 for peptides from B) histatin-1 and D) HSA. Shown are the data for investigated peptides (squares) and GGH (diamonds), either for experiments with nonphosphorylated (blue, full symbols) or phosphorylated versions (red, open symbols).

Interestingly, copper affinities of nonphosphorylated DAHKSE-am and DSHEKR-am differ substantially from each other. Nevertheless, the value for DAHKSE-am is close to that obtained recently for human serum albumin whole protein (log *K*_f_ = 13.02 ± 0.05).^2^ The affinity for DSHEKR-am, in turn, is equal to the one for DTHFPI-am, the N-terminal motif of hepcidin, claimed to be the strongest Cu(II) chelator among ATCUN motifs.^10^ Both peptides have an Asp residue in the first position and a Ser or Thr residue in the second. The acidic amino acid residue in the first position and the hydroxyl-containing one in the second position presumably ensure such tight copper binding.

Human histatin-1 and human serum albumin are phosphorylated on serine residues tested in this work.^11−13^ However, about 20% of human histatin-1 is nonphosphorylated on Ser2.^14,15^ Although phosphorylation of histatin-1 is beneficial for its binding to hydroxyapatite,^16^ this peptide plays many other roles, e.g., promotes angiogenesis^17^ and exhibits antimicrobial activity.^3,18^ The involvement of phosphorylation in those processes is not known yet. The binding of metal ions by antimicrobial peptides leads to sequestration of this metal from pathogens or is necessary for other antimicrobial modes of action.^18^ Contribution of copper to maintaining microbial oral hygiene is probable as there is an inverse relationship of caries status and Cu(II) concentration in the saliva of children.^19^ Overall, Ser2 phosphorylation may affect metal-related functions of histatin-1.

Human serum albumin was found to be phosphorylated on Ser5 by Fam20C kinase.^13^ However, the extent of this phosphorylation is not known. Although only 2% of ATCUN in HSA is occupied by Cu(II) ions in blood plasma,^20^ such saturation is not known for other parts of the organism. Most of HSA (60–70%) is in the extravascular pool,^21^ e.g., in skin, saliva, and cerebrospinal fluid.^22^ Both saturation with Cu(II) ions and phosphorylation on Ser5 may differ dramatically from the one in the blood plasma. Such post-translational modification may regulate Cu(II) binding by HSA locally or transiently.

The influence of amino acid residue type on the stability of the Cu(II) complex with the ATCUN motif has been thoroughly studied. One of the most evident rules described in the literature is the inverse linear dependence of the affinity to metal ion on the basicity of the N-terminal amine.^23,24^ There is also a hypothesis that the same dependence is valid for amide nitrogens.^4^ Interestingly, the presence of two Asp residues in the ATCUN motif (Asp-Asp-His) is correlated with the highest basicity of the N-terminal amine and, consequently, with the lowest affinity to Cu(II).^24^ The basicity of amine in the free serine amino acid is also heightened by phosphorylation of its side chain (9.85 vs 9.25).^25,26^ Therefore, the results presented in this work suggest that phosphate moiety, similarly acidic as the Asp side chain, may weaken the binding of Cu(II) ions by stabilizing the protonated state of the amine and amide nitrogens.

Based on the recent analysis of N-terminal sequences in human proteins,^1^ the selection of human proteins with the ATCUN motif and serine or threonine residue within the first five positions was performed and presented in Table S1. The threonine residue was added to this analysis as its phosphorylation probably shows the same impact on Cu(II) binding as serine modification. There are 65 human proteins, either secreted or with N-terminus exposed on the extracellular side of the cell membrane, with ATCUN motifs containing at least one Ser or Thr (Table S1). Seven proteins with such sequences are localized in the endoplasmic reticulum or Golgi apparatus. It sums up to 72 proteins localized in relatively oxidizing compartments, thus, with a higher probability to meet divalent copper ions.^1^ Interestingly, more than 100 other proteins are localized in compartments with a more reducing state (Table S1), such as cytoplasm, nucleus, or mitochondrion.^1^ The binding of Cu(II) by those proteins is questionable (because intracellularly Cu(I) is more prevalent than Cu(II)) but cannot be excluded that may occur transiently or in pathological states. The frequency of Ser or Thr in the sequence position 1, 2, 4, or 5 throughout the whole selection (223 proteins, Table S1) is relatively uniform with a slight prevalence of serine residues (Figure S7).

The results presented in this work indicate that phosphorylation of serine residues markedly weakens the stability of Cu(II) complexes with the peptides comprising N-terminal sequences of human histatin-1 and human serum albumin. Thus, phosphorylation is perhaps a mechanism of fine-tuning the metal-related functions of these proteins. It may also be a general mechanism valid for other proteins or peptides with ATCUN motifs.

## Supporting Information Available

Experimental section; details related to decomposition of CD spectra and determination of Cu(II) binding affinity; and list of human proteins with ATCUN motif and serine or threonine residue within first five positions (PDF)
