# Supplementary material for: Phosphorylation Impacts Cu(II) Binding by ATCUN Motifs
Source: Inorg Chem. 2021 Jun 7;60(12):8447–50. doi: 10.1021/acs.inorgchem.1c00939 (PMC8277166; doi:10.1021/acs.inorgchem.1c00939)
Supplement: Supplementary file 1 — ic1c00939_si_001.pdf [file ic1c00939_si_001.pdf]

Supporting Information

Phosphorylation impacts Cu(II) binding by ATCUN motifs

Tomasz Frączyk *

Institute of Biochemistry and Biophysics, Polish Academy of Sciences, Pawińskiego 5a, 02-106 Warsaw, Poland

* Email: tfraczyk@ibb.waw.pl (T. Frączyk)

Experimental section

Materials

The hexapeptides were purchased from GenScript. All other chemicals were from Sigma Aldrich.

Methods

The UV-vis and CD spectra were collected using a Lambda 950 spectrophotometer (PerkinElmer) and a J-815 CD spectrometer (JASCO), respectively.

Standard deviation (±SD) was shown for each calculated parameter.

Concentrations determination

The concentration of each peptide was determined by the titration at pH 7.4 (50 mM HEPES) with $CuCl_2$ solution (with known concentration) with UV-vis (at 380, 525, and 750 nm) and CD (at 490 and 570 nm, for hexapeptides or 497 and 582 nm for GGH) detection. Thus, appearing *d–d* bands (around 500–600 nm) for Cu(peptide) or turbidity (at 380 and 750 nm) for $Cu(OH)_2$ were analyzed to find globally the inflection points in titration plots (Fig. S1).

Calculation of spectra of each complex

The spectra (shown below) for each Cu(peptide) 1:1 genuine complex were calculated from the above-described concentration determination experiments. The spectra were then used for spectra decomposition of hexapeptides and GGH mixtures competing for Cu(II) ions (Fig. S2).

Calculation of pK_a values for the formation of 4N copper complexes

The values of pK_a shown in this work are equal to the pH value at which half of the total concentration of copper is in 4N square planar complexes with hexapeptides. Such values were obtained from spectroscopic pH-metric titrations by fitting the data from UV-vis and CD spectroscopies to the equation:

$$S = \frac{S_{low} + S_{high} \times 10^{n \times (pH - pKa)}}{1 + 10^{n \times (pH - pKa)}} \quad (1)$$

where S is signal (either absorbance or ellipticity) at a specific wavelength, S_{low} and S_{high} are signals at low pH (no binding) and high pH (saturation of binding), respectively, *n* is the Hill coefficient, and pH is the pH value at which the signal (S) was registered. The pK_a values were fitted globally for absorbance and ellipticity.

Decomposition of the spectra

The decompositions were performed by iterative weighted additions of the spectra of Cu(hexapeptide) and Cu(GGH) spectra to minimize the root-mean-square deviation between the reconstructed and the experimental spectrum (Fig. S3-S6). The procedure was performed with the use of the Solver tool in Microsoft Excel.

Determination of Cu(II) binding strength of investigated peptides

The formation constants for hexapeptides and GGH are:

$$K_{f(Cu(hexapeptide))} = \frac{[Cu(hexapeptide)]}{[Cu][hexapeptide]}; \qquad \text{Cu + hexapeptide} \rightleftarrows \text{Cu(hexapeptide)} \qquad (2)$$

$$K_{f(Cu(GGH))} = \frac{[Cu(GGH)]}{[Cu][GGH]}; \qquad \text{Cu + GGH} \rightleftarrows \text{Cu(GGH)} \qquad (3)$$

while the total concentrations of all forms of hexapeptide and GGH are:

$[hexapeptide]_T$ = [Cu(hexapeptide)] + [hexapeptide] (4)

$[GGH]_T$ = [Cu(GGH)] + [GGH] (5)

Combining equations 1 – 4, the ratio of the binding constants can be expressed as:

$$\frac{K_{f(Cu(hexapeptide))}}{K_{f(Cu(GGH))}} = \frac{[Cu(hexapeptide)]([GGH]_T - [Cu(GGH)])}{[Cu(GGH)]([hexapeptide]_T - [Cu(hexapeptide)])} \qquad (6)$$

$[hexapeptide]_T$, $[GGH]_T$, and $K_{f(Cu(GGH))}$ ($10^{12.215}$ M^{-1}; ChemBioChem 2020; 21: 331-334) are known. [Cu(hexapeptide)] and [Cu(GGH)] are found by the above-described decomposition of CD spectra of Cu/hexapeptide/GGH mixtures. The relative Cu(II) binding strength (at pH 7.4) of investigated hexapeptides and GGH can be determined from the linear fit to data on a plot of [Cu(hexapeptide)]($[GGH]_T$ – [Cu(GGH)]) versus [Cu(GGH)]($[hexapeptide]_T$ – [Cu(hexapeptide)]). The slope of this dependence is equal to the number saying how many times the formation constant (K_f) for Cu(hexapeptide) is higher than for Cu(GGH) (Figure 5A and C, main text).

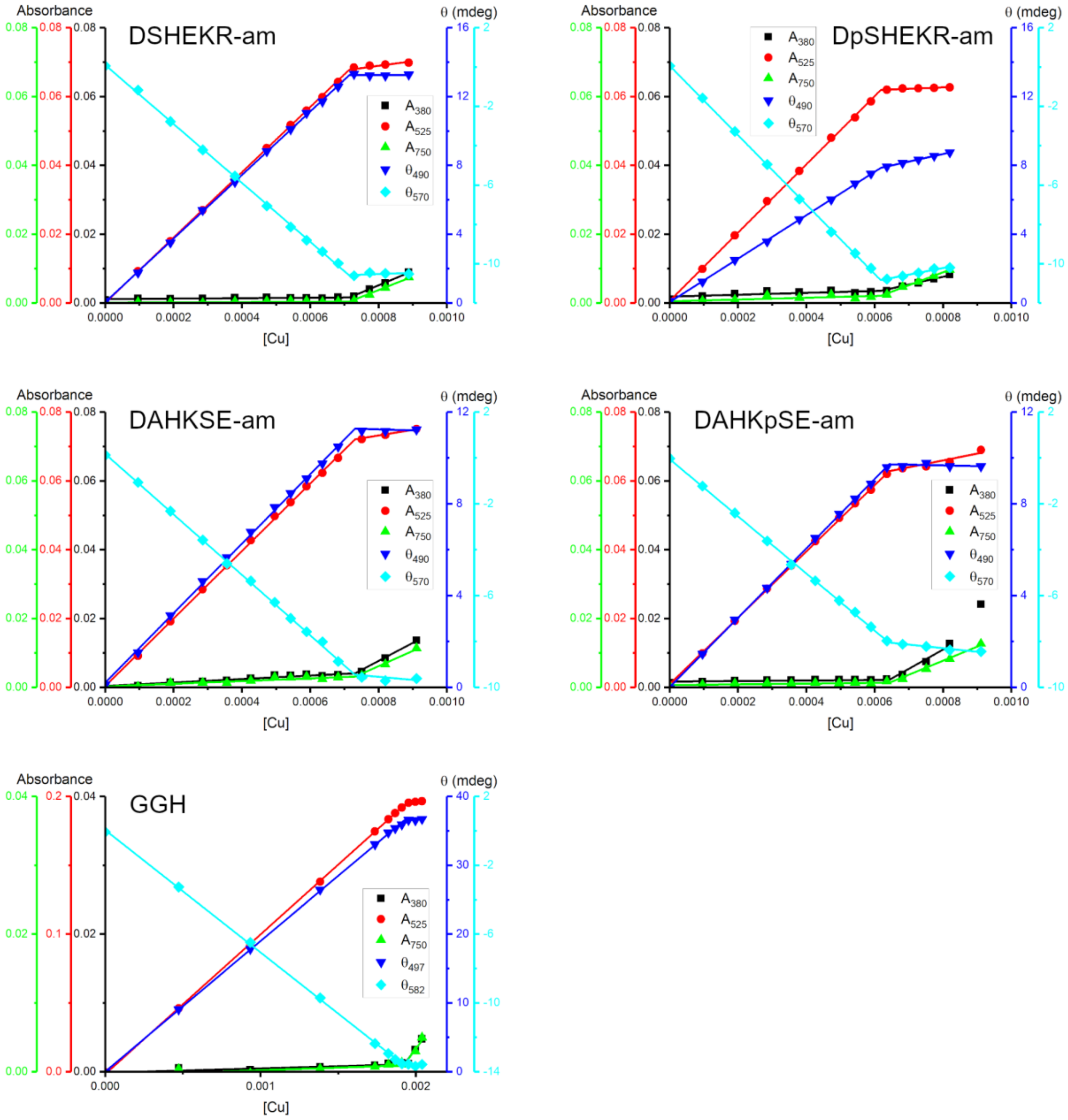

Figure S1. The dependence of absorbance and CD signal on Cu(II) concentration for Cu/peptide mixtures (pH 7.4).

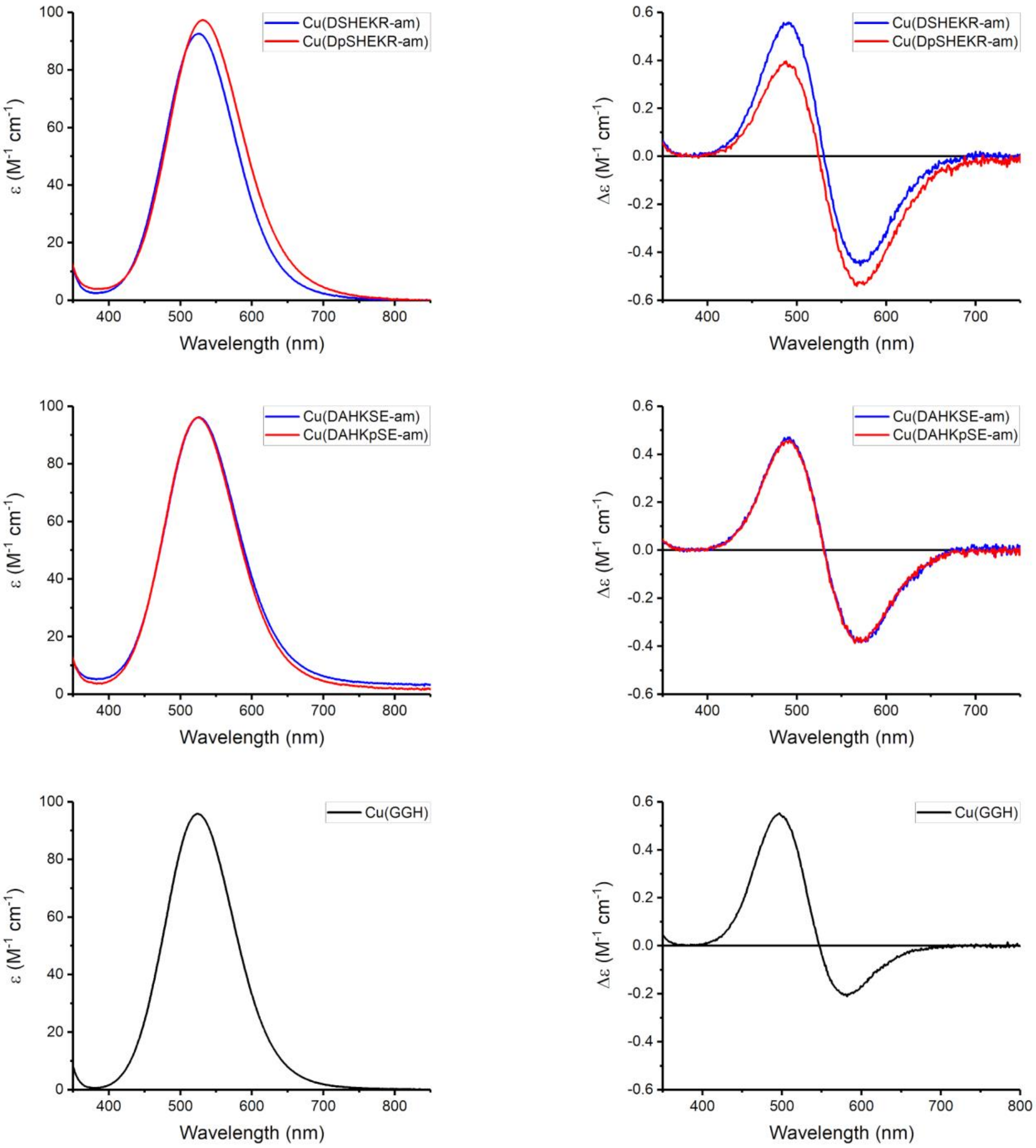

Figure S2. The spectra of Cu(peptide) 1:1 genuine complexes (pH 7.4).

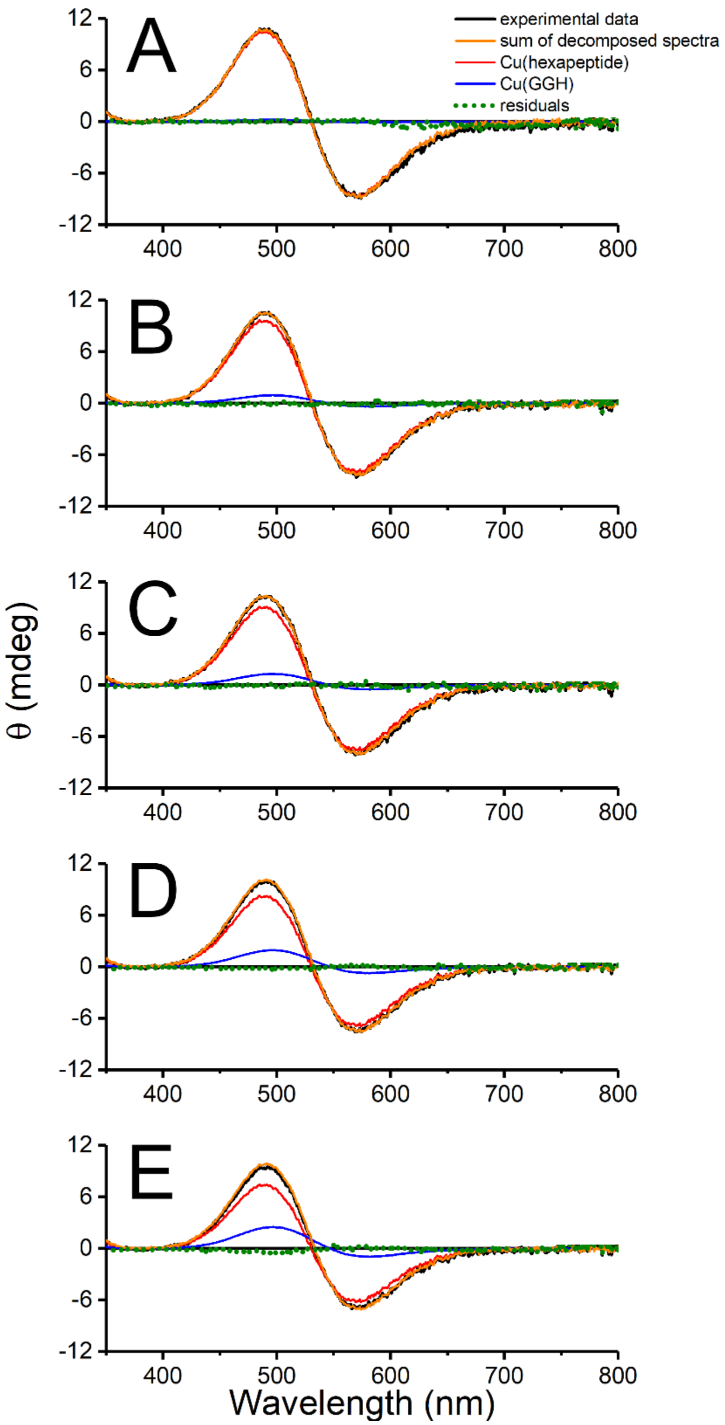

Figure S3. The decomposition of components spectra. The mixture contained 50 mM HEPES (pH 7.4), 0.6 mM $CuCl_2$, 0.67 mM DSHEKR-am, and increasing concentrations of GGH: 2, 5, 8, 12, and 16 mM, for A, B, C, D, and E, respectively. The corresponding percentage of each species is shown in Figure 5B (main text).

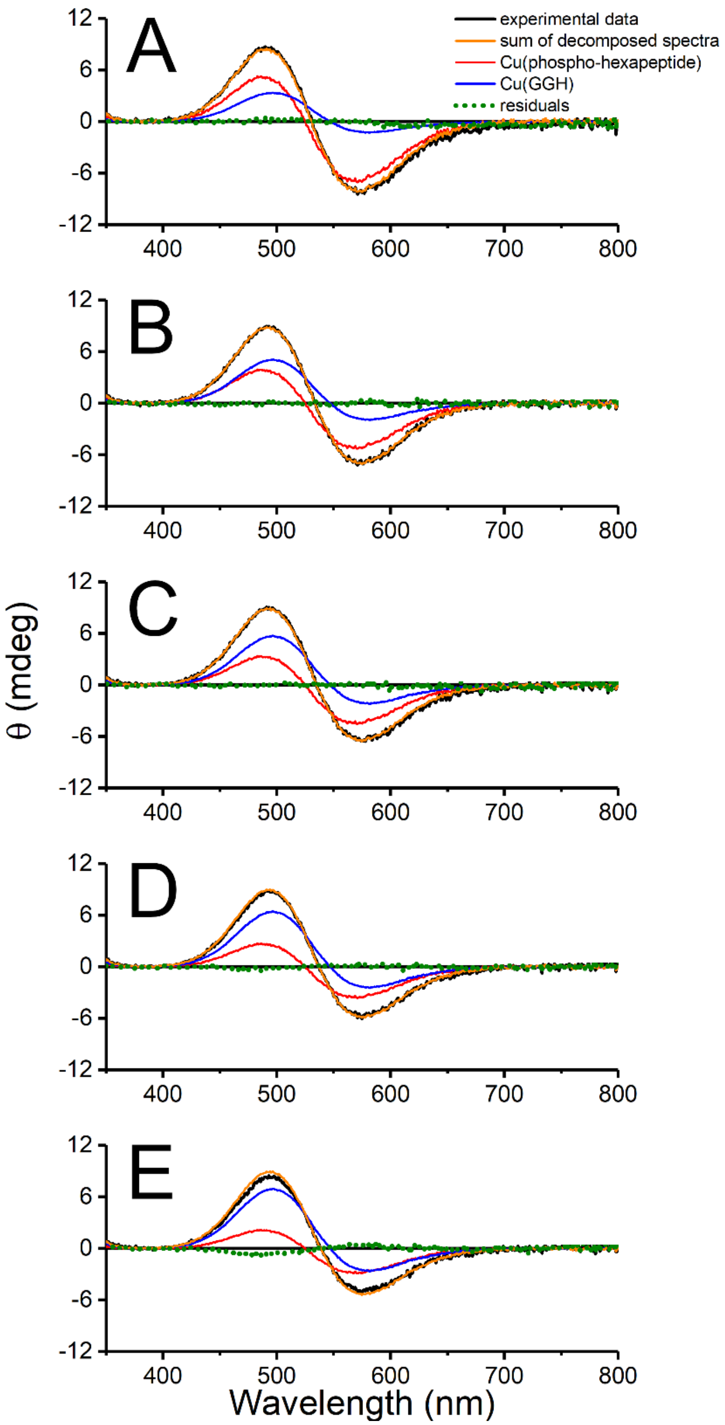

Figure S4. The decomposition of components spectra. The mixture contained 50 mM HEPES (pH 7.4), 0.6 mM $CuCl_2$, 0.67 mM DpSHEKR-am, and increasing concentrations of GGH: 2, 5, 8, 12, and 16 mM, for A, B, C, D, and E, respectively. The corresponding percentage of each species is shown in Figure 5B (main text).

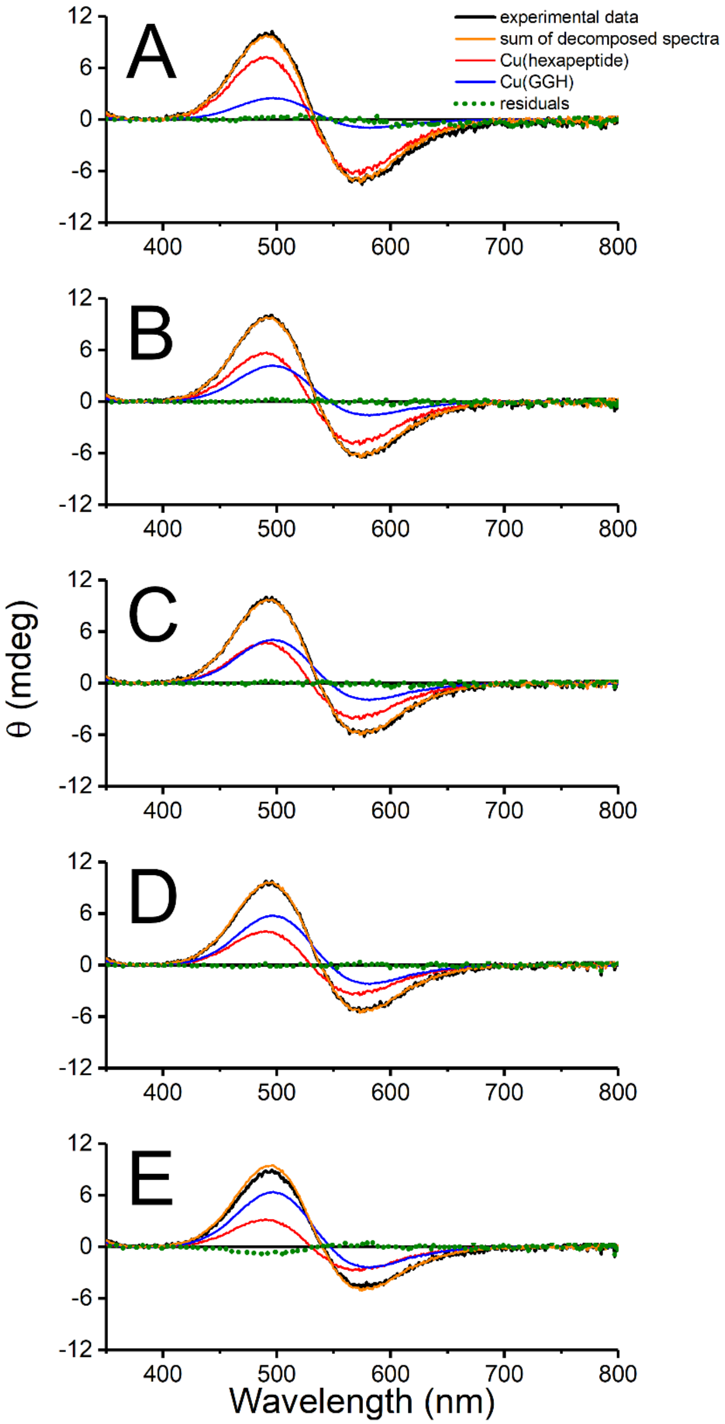

Figure S5. The decomposition of components spectra. The mixture contained 50 mM HEPES (pH 7.4), 0.6 mM $CuCl_2$, 0.67 mM DAHKSE-am, and increasing concentrations of GGH: 2, 5, 8, 12, and 16 mM, for A, B, C, D, and E, respectively. The corresponding percentage of each species is shown in Figure 5D (main text).

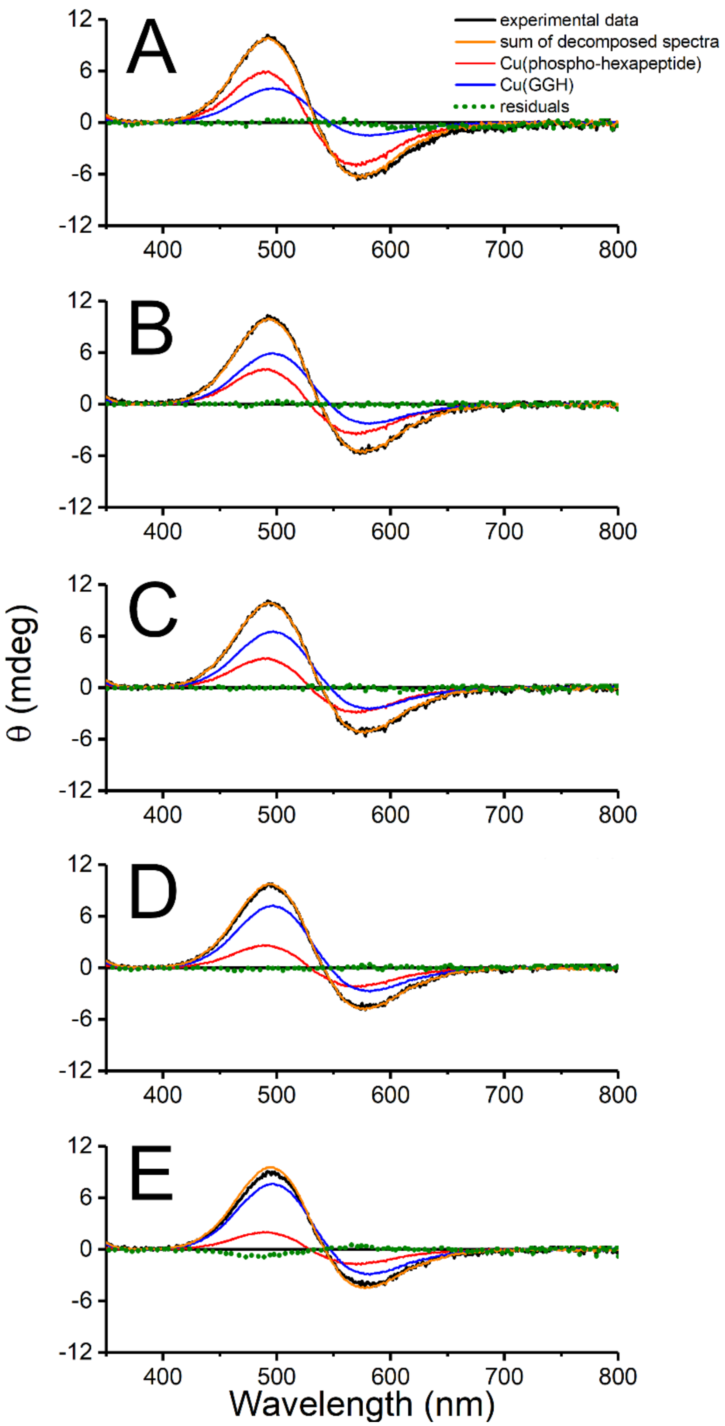

Figure S6. The decomposition of components spectra. The mixture contained 50 mM HEPES (pH 7.4), 0.6 mM $CuCl_2$, 0.67 mM DAHKpSE-am, and increasing concentrations of GGH: 2, 5, 8, 12, and 16 mM, for A, B, C, D, and E, respectively. The corresponding percentage of each species is shown in Figure 5D (main text).

Table S1. The list of human proteins with ATCUN motif and serine or threonine residue within the first five positions.[a,b]

	UniProtKB entry identifier	Entry name	Protein name	Gene name	First 5 aa	Notes	Subcellular location
1	P15516	HIS3_HUMAN	**Histatin-3**	HTN3	DSHAK		Secreted
2	P15515	HIS1_HUMAN	**Histatin-1**	HTN1	DSHEK	phospho-Ser (Uniprot)	
3	Q16663	CCL15_HUMAN	**C-C motif chemokine 15**	CCL15	SFHFA		
4	Q96LR4	TAFA4_HUMAN	**Chemokine-like protein TAFA-4**	TAFA4	SQHLR		
5	Q9H293	IL25_HUMAN	**Interleukin-25**	IL25	YSHWP		
6	P20827	EFNA1_HUMAN	**Ephrin-A1**	EFNA1	DRHTV		
7	O95813	CER1_HUMAN	**Cerberus**	CER1	TRHQD		
8	Q9GZP0	PDGFD_HUMAN	**Platelet-derived growth factor D**	PDGFD	SYHDR		
9	P04279	SEMG1_HUMAN	**Semenogelin-1**	SEMG1	TYHVD		
10	P14222	PERF_HUMAN	**Perforin-1**	PRF1	PCHTA		
11	Q5XG92	EST4A_HUMAN	**Carboxylesterase 4A**	CES4A	ALHTK		
12	P02768	ALBU_HUMAN	**Albumin**	ALB	DAHKS	phospho-Ser (Uniprot)	
13	P39060	COIA1_HUMAN	**Collagen alpha-1(XVIII) chain**	COL18A1	HSHRD		
14	Q75N90	FBN3_HUMAN	**Fibrillin-3**	FBN3	SAHRD		Extracellular matrix or space
15	P35556	FBN2_HUMAN	**Fibrillin-2**	FBN2	SIHEP		
16	P98160	PGBM_HUMAN	**Basement membrane-specific heparan ...**	HSPG2	VTHGL		
17	Q86UW2	OSTB_HUMAN	**Organic solute transporter subunit ...**	SLC51B	MEHSE		Extracellular side of cell membrane
18	C9JQL5	DSA2D_HUMAN	**Putative dispanin subfamily A membe...**		MNHTV		
19	O15431	COPT1_HUMAN	**High affinity copper uptake protein...**	SLC31A1	MDHSH		
20	P33681	CD80_HUMAN	**T-lymphocyte activation antigen CD8...**	CD80	VIHVT		
21	Q8NGZ3	O13G1_HUMAN	**Olfactory receptor 13G1**	OR13G1	MNHSV		
22	Q9NZP0	OR6C3_HUMAN	**Olfactory receptor 6C3**	OR6C3	MNHTM		
23	Q8NGD4	OR4K1_HUMAN	**Olfactory receptor 4K1**	OR4K1	MAHTN		
24	Q96RD2	O52B2_HUMAN	**Olfactory receptor 52B2**	OR52B2	MSHTN		
25	Q8NGI3	O56B1_HUMAN	**Olfactory receptor 56B1**	OR56B1	MNHMS		
26	Q8NH55	O52E5_HUMAN	**Olfactory receptor 52E5**	OR52E5	MLHTN		
27	P29016	CD1B_HUMAN	**T-cell surface glycoprotein CD1b**	CD1B	SEHAF		
28	P17693	HLAG_HUMAN	**HLA class I histocompatibility anti...**	HLA-G	GSHSM		
29	Q95460	HMR1_HUMAN	**Major histocompatibility complex cl...**	MR1	RTHSL		

30	P30511	HLAF_HUMAN	**HLA class I histocompatibility anti...**	HLA-F	GSHSL		
31	Q6DWJ6	GP139_HUMAN	**Probable G-protein coupled receptor...**	GPR139	MEHTH		
32	P13747	HLAE_HUMAN	**HLA class I histocompatibility anti...**	HLA-E	GSHSL		
33	P01893	HLAH_HUMAN	**Putative HLA class I histocompatibi...**	HLA-H	RSHSM		
34	P01889	HLAB_HUMAN	**HLA class I histocompatibility anti...**	HLA-B	GSHSM		
35	P04439	HLAA_HUMAN	**HLA class I histocompatibility anti...**	HLA-A	GSHSM		
36	P10321	HLAC_HUMAN	**HLA class I histocompatibility anti...**	HLA-C	CSHSM		
37	Q5VW32	BROX_HUMAN	**BRO1 domain-containing protein BROX**	BROX	MTHWF		
38	P23416	GLRA2_HUMAN	**Glycine receptor subunit alpha-2**	GLRA2	KDHDS		
39	P02708	ACHA_HUMAN	**Acetylcholine receptor subunit alph...**	CHRNA1	SEHET		
40	A0A1B0GV85	RELD1_HUMAN	**Reelin domain-containing protein 1**	REELD1	FSHGA		
41	A6NGU5	GGT3_HUMAN	**Putative glutathione hydrolase 3 pr...**	GGT3P	TAHLS		
42	P19440	GGT1_HUMAN	**Glutathione hydrolase 1 proenzyme**	GGT1	TAHLS		
43	P36269	GGT5_HUMAN	**Glutathione hydrolase 5 proenzyme**	GGT5	TSHVS		
44	Q13586	STIM1_HUMAN	**Stromal interaction molecule 1**	STIM1	LSHSH		
45	Q16549	PCSK7_HUMAN	**Proprotein convertase subtilisin/ke...**	PCSK7	SVHFN		
46	Q8IVU1	IGDC3_HUMAN	**Immunoglobulin superfamily DCC subc...**	IGDCC3	LGHSA		
47	Q13002	GRIK2_HUMAN	**Glutamate receptor ionotropic, kain...**	GRIK2	TTHVL		
48	Q9Y5H7	PCDA5_HUMAN	**Protocadherin alpha-5**	PCDHA5	QLHYS		
49	Q9UN72	PCDA7_HUMAN	**Protocadherin alpha-7**	PCDHA7	QLHYS		
50	Q9UN75	PCDAC_HUMAN	**Protocadherin alpha-12**	PCDHA12	QLHYS		
51	Q9UN74	PCDA4_HUMAN	**Protocadherin alpha-4**	PCDHA4	QLHYS		
52	Q9Y5I2	PCDAA_HUMAN	**Protocadherin alpha-10**	PCDHA10	QLHYS		
53	Q9Y5I1	PCDAB_HUMAN	**Protocadherin alpha-11**	PCDHA11	QLHYS		
54	Q9Y5H8	PCDA3_HUMAN	**Protocadherin alpha-3**	PCDHA3	QLHYS		
55	Q9Y5H6	PCDA8_HUMAN	**Protocadherin alpha-8**	PCDHA8	QLHYS		
56	Q9Y5H5	PCDA9_HUMAN	**Protocadherin alpha-9**	PCDHA9	QLHYS		
57	Q9Y5I0	PCDAD_HUMAN	**Protocadherin alpha-13**	PCDHA13	QLHYS		
58	Q9UN73	PCDA6_HUMAN	**Protocadherin alpha-6**	PCDHA6	QLHYS		
59	Q9Y5I3	PCDA1_HUMAN	**Protocadherin alpha-1**	PCDHA1	QLHYS		
60	Q9P2E7	PCD10_HUMAN	**Protocadherin-10**	PCDH10	QLHYT		
61	Q6IEE7	T132E_HUMAN	**Transmembrane protein 132E**	TMEM132E	RSHPA		
62	P01133	EGF_HUMAN	**Pro-epidermal growth factor**	EGF	PQHWS		

63	P15941	MUC1_HUMAN	**Mucin-1**	MUC1	SGHAS		
64	Q6P1J6	PLB1_HUMAN	**Phospholipase B1, membrane-associat...**	PLB1	QIHTS		
65	Q9BZM4	ULBP3_HUMAN	**UL16-binding protein 3**	ULBP3	DAHSL		
66	P52961	NAR1_HUMAN	**GPI-linked NAD(P)(+)--arginine ADP-...**	ART1	QSHPI		Endoplasmic reticulum / Golgi apparatus
67	Q5SYC1	CLVS2_HUMAN	**Clavesin-2**	CLVS2	MTHLQ		
68	Q58HT5	AWAT1_HUMAN	**Acyl-CoA wax alcohol acyltransferas...**	AWAT1	MAHSK		
69	Q9Y2T2	AP3M1_HUMAN	**AP-3 complex subunit mu-1**	AP3M1	MIHSL		
70	P53677	AP3M2_HUMAN	**AP-3 complex subunit mu-2**	AP3M2	MIHSL		
71	Q96CV9	OPTN_HUMAN	**Optineurin**	OPTN	MSHQP		
72	Q4ZIN3	MBRL_HUMAN	**Membralin**	TMEM259	SEHVE	N-acetyl-Ser (Uniprot)	
73	Q9UBJ2	ABCD2_HUMAN	**ATP-binding cassette sub-family D m...**	ABCD2	MTHML		Peroxisome
74	Q92609	TBCD5_HUMAN	**TBC1 domain family member 5**	TBC1D5	MYHSL		Endosome
75	A6NGE4	DC8L1_HUMAN	**DDB1- and CUL4-associated factor 8-...**	DCAF8L1	MSHQE		Cytoplasm
76	P0C7V8	DC8L2_HUMAN	**DDB1- and CUL4-associated factor 8-...**	DCAF8L2	MSHQE		
77	Q96NH3	BROMI_HUMAN	**Protein broad-minded**	TBC1D32	MAHFS		
78	P62861	RS30_HUMAN	**40S ribosomal protein S30**	FAU	KVHGS		Cytosol
79	Q8IUB9	KR191_HUMAN	**Keratin-associated protein 19-1**	KRTAP19-1	MSHYG		
80	P59990	KR121_HUMAN	**Keratin-associated protein 12-1**	KRTAP12-1	MCHTS		
81	P60328	KR123_HUMAN	**Keratin-associated protein 12-3**	KRTAP12-3	MCHTS		
82	P60329	KR124_HUMAN	**Keratin-associated protein 12-4**	KRTAP12-4	MCHTS		
83	Q8NHR9	PROF4_HUMAN	**Profilin-4**	PFN4	MSHLQ		
84	P46779	RL28_HUMAN	**60S ribosomal protein L28**	RPL28	SAHLQ	N-acetyl-Ser (Uniprot)	
85	P59991	KR122_HUMAN	**Keratin-associated protein 12-2**	KRTAP12-2	MCHTS		
86	Q9BYQ2	KRA94_HUMAN	**Keratin-associated protein 9-4**	KRTAP9-4	MTHCC		
87	Q9BYP9	KRA99_HUMAN	**Keratin-associated protein 9-9**	KRTAP9-9	MTHCC		
88	Q9BYQ3	KRA93_HUMAN	**Keratin-associated protein 9-3**	KRTAP9-3	MTHCC		
89	Q9BYQ0	KRA98_HUMAN	**Keratin-associated protein 9-8**	KRTAP9-8	MTHCC		
90	A8MVA2	KRA96_HUMAN	**Keratin-associated protein 9-6**	KRTAP9-6	MTHCC		
91	A8MTY7	KRA97_HUMAN	**Keratin-associated protein 9-7**	KRTAP9-7	MTHCC		
92	Q9BYQ4	KRA92_HUMAN	**Keratin-associated protein 9-2**	KRTAP9-2	MTHCC		
93	A8MXZ3	KRA91_HUMAN	**Keratin-associated protein 9-1**	KRTAP9-1	MTHCC		

94	P00918	CAH2_HUMAN	**Carbonic anhydrase 2**	CA2	SHHWG	N-acetyl-Ser; phospho-Ser (Uniprot)	
95	Q15417	CNN3_HUMAN	**Calponin-3**	CNN3	MTHFN		
96	Q9Y5B8	NDK7_HUMAN	**Nucleoside diphosphate kinase 7**	NME7	MNHSE		
97	Q9P0J7	KCMF1_HUMAN	**E3 ubiquitin-protein ligase KCMF1**	KCMF1	SRHEG	N-acetyl-Ser; phospho-Ser (Uniprot)	
98	Q92901	RL3L_HUMAN	**60S ribosomal protein L3-like**	RPL3L	MSHRK		
99	Q9HCP0	KC1G1_HUMAN	**Casein kinase I isoform gamma-1**	CSNK1G1	MDHPS		
100	P08729	K2C7_HUMAN	**Keratin, type II cytoskeletal 7**	KRT7	SIHFS	N-acetyl-Ser; phospho-Ser (Uniprot)	
101	P41219	PERI_HUMAN	**Peripherin**	PRPH	MSHHP		
102	P23381	SYWC_HUMAN	**Tryptophan--tRNA ligase, cytoplasmi...**	WARS1	SNHGP		
103	Q7Z794	K2C1B_HUMAN	**Keratin, type II cytoskeletal 1b**	KRT77	MSHQF		
104	Q6ZVT0	TTL10_HUMAN	**Inactive polyglycylase TTLL10**	TTLL10	MDHSC		
105	Q8IYB7	DI3L2_HUMAN	**DIS3-like exonuclease 2**	DIS3L2	MSHPD		
106	P35573	GDE_HUMAN	**Glycogen debranching enzyme**	AGL	MGHSK		
107	Q9Y566	SHAN1_HUMAN	**SH3 and multiple ankyrin repeat dom...**	SHANK1	MTHSP		
108	Q01628	IFM3_HUMAN	**Interferon-induced transmembrane pr...**	IFITM3	MNHTV		Cytoplasmic side
109	P46695	IEX1_HUMAN	**Radiation-inducible immediate-early...**	IER3	MCHSR		
110	Q6GV28	TM225_HUMAN	**Transmembrane protein 225**	TMEM225	MVHVS		
111	O95070	YIF1A_HUMAN	**Protein YIF1A**	YIF1A	AYHSG	N-acetyl-Ala (Uniprot)	
112	P15907	SIAT1_HUMAN	**Beta-galactoside alpha-2,6-sialyltr...**	ST6GAL1	MIHTN		
113	O95528	GTR10_HUMAN	**Solute carrier family 2, facilitate...**	SLC2A10	MGHSP		
114	Q8TDI7	TMC2_HUMAN	**Transmembrane channel-like protein ...**	TMC2	MSHQV		
115	Q6P179	ERAP2_HUMAN	**Endoplasmic reticulum aminopeptidas...**	ERAP2	MFHSS		
116	Q9BYT9	ANO3_HUMAN	**Anoctamin-3**	ANO3	MVHHS		
117	P04554	PRM2_HUMAN	**Protamine-2**	PRM2	RTHGQ		Nucleus
118	Q86WQ0	NR2CA_HUMAN	**Nuclear receptor 2C2-associated pro...**	NR2C2AP	MTHSL		
119	P78543	BTG2_HUMAN	**Protein BTG2**	BTG2	MSHGK		
120	Q9UBY9	HSPB7_HUMAN	**Heat shock protein beta-7**	HSPB7	MSHRT		
121	P56179	DLX6_HUMAN	**Homeobox protein DLX-6**	DLX6	MSHSQ		
122	P49703	ARL4D_HUMAN	**ADP-ribosylation factor-like protei...**	ARL4D	GNHLT	N-myristoyl Gly (Uniprot)	
123	Q9GZP4	PITH1_HUMAN	**PITH domain-containing protein 1**	PITHD1	MSHGH		

124	Q5JUR7	TEX30_HUMAN	**Testis-expressed protein 30**	TEX30	MSHTE		
125	P60008	HILS1_HUMAN	**Spermatid-specific linker histone H...**	HILS1	MLHAS		
126	O14595	CTDS2_HUMAN	**Carboxy-terminal domain RNA polymer...**	CTDSP2	MEHGS	phospho-Ser (Uniprot)	
127	Q15744	CEBPE_HUMAN	**CCAAT/enhancer-binding protein epsi...**	CEBPE	MSHGT		
128	Q15072	OZF_HUMAN	**Zinc finger protein OZF**	ZNF146	MSHLS		
129	Q9P0W2	HM20B_HUMAN	**SWI/SNF-related matrix-associated a...**	HMG20B	MSHGP		
130	P51946	CCNH_HUMAN	**Cyclin-H**	CCNH	MYHNS	phospho-Ser (Uniprot)	
131	Q12952	FOXL1_HUMAN	**Forkhead box protein L1**	FOXL1	MSHLF		
132	Q96DB2	HDA11_HUMAN	**Histone deacetylase 11**	HDAC11	MLHTT		
133	Q9NS37	ZHANG_HUMAN	**CREB/ATF bZIP transcription factor**	CREBZF	MRHSL		
134	O15143	ARC1B_HUMAN	**Actin-related protein 2/3 complex s...**	ARPC1B	AYHSF		
135	Q99733	NP1L4_HUMAN	**Nucleosome assembly protein 1-like ...**	NAP1L4	ADHSF	N-acetyl-Ala; phospho-Ser (Uniprot)	
136	A4D1E9	GTPBA_HUMAN	**GTP-binding protein 10**	GTPBP10	MVHCS		
137	Q96LI6	HSFY1_HUMAN	**Heat shock transcription factor, Y-...**	HSFY1	MAHVS		
138	Q6NYC1	JMJD6_HUMAN	**Bifunctional arginine demethylase a...**	JMJD6	MNHKS		
139	P09086	PO2F2_HUMAN	**POU domain, class 2, transcription ...**	POU2F2	MVHSS		
140	Q9HAZ1	CLK4_HUMAN	**Dual specificity protein kinase CLK...**	CLK4	MRHSK		
141	P49759	CLK1_HUMAN	**Dual specificity protein kinase CLK...**	CLK1	MRHSK		
142	Q75N03	HAKAI_HUMAN	**E3 ubiquitin-protein ligase Hakai**	CBLL1	MDHTD		
143	Q06330	SUH_HUMAN	**Recombining binding protein suppres...**	RBPJ	MDHTE		
144	A2A288	ZC12D_HUMAN	**Probable ribonuclease ZC3H12D**	ZC3H12D	MEHPS		
145	Q9HCL3	ZFP14_HUMAN	**Zinc finger protein 14 homolog**	ZFP14	MAHGS		
146	P19532	TFE3_HUMAN	**Transcription factor E3**	TFE3	MSHAA		
147	Q8NHQ9	DDX55_HUMAN	**ATP-dependent RNA helicase DDX55**	DDX55	MEHVT		
148	Q9Y2D9	ZN652_HUMAN	**Zinc finger protein 652**	ZNF652	MSHTA		
149	Q93052	LPP_HUMAN	**Lipoma-preferred partner**	LPP	MSHPS		
150	Q86TM3	DDX53_HUMAN	**Probable ATP-dependent RNA helicase...**	DDX53	MSHWA		
151	O75290	Z780A_HUMAN	**Zinc finger protein 780A**	ZNF780A	MVHGS		
152	Q9NXZ2	DDX43_HUMAN	**Probable ATP-dependent RNA helicase...**	DDX43	MSHHG		
153	Q9NVP1	DDX18_HUMAN	**ATP-dependent RNA helicase DDX18**	DDX18	MSHLP		
154	Q96EF0	MTMR8_HUMAN	**Myotubularin-related protein 8**	MTMR8	MDHIT		
155	Q8TDB6	DTX3L_HUMAN	**E3 ubiquitin-protein ligase DTX3L**	DTX3L	ASHLR	N-acetyl-Ala (Uniprot)	

156	Q13835	PKP1_HUMAN	**Plakophilin-1**	PKP1	MNHSP		
157	Q14872	MTF1_HUMAN	**Metal regulatory transcription fact...**	MTF1	GEHSP	N-acetyl-Gly; phospho-Ser (Uniprot)	
158	Q8TBE0	BAHD1_HUMAN	**Bromo adjacent homology domain-cont...**	BAHD1	MTHTR		
159	Q14694	UBP10_HUMAN	**Ubiquitin carboxyl-terminal hydrola...**	USP10	ALHSP	N-acetyl-Ala (Uniprot)	
160	Q9Y6R6	Z780B_HUMAN	**Zinc finger protein 780B**	ZNF780B	MVHGS		
161	Q8NB50	ZFP62_HUMAN	**Zinc finger protein 62 homolog**	ZFP62	MSHLK		
162	P48552	NRIP1_HUMAN	**Nuclear receptor-interacting protei...**	NRIP1	MTHGE		
163	Q14678	KANK1_HUMAN	**KN motif and ankyrin repeat domain-...**	KANK1	MAHTT		
164	Q12769	NU160_HUMAN	**Nuclear pore complex protein Nup160**	NUP160	MLHLS		
165	Q9NTI5	PDS5B_HUMAN	**Sister chromatid cohesion protein P...**	PDS5B	MAHSK		
166	Q96L91	EP400_HUMAN	**E1A-binding protein p400**	EP400	MHHGT		
167	Q03164	KMT2A_HUMAN	**Histone-lysine N-methyltransferase ...**	KMT2A	MAHSC		
168	Q9NRX2	RM17_HUMAN	**39S ribosomal protein L17, mitochon...**	MRPL17	ISHGR		Mitochondrion
169	O95169	NDUB8_HUMAN	**NADH dehydrogenase [ubiquinone] 1 b...**	NDUFB8	ASHMT		
170	P00414	COX3_HUMAN	**Cytochrome c oxidase subunit 3**	MT-CO3	MTHQS		
171	P45880	VDAC2_HUMAN	**Voltage-dependent anion-selective c...**	VDAC2	ATHGQ	N-acetyl-Ala (Uniprot)	
172	Q9H1K4	GHC2_HUMAN	**Mitochondrial glutamate carrier 2**	SLC25A18	MTHQD		
173	Q9HB07	MYG1_HUMAN	**UPF0160 protein MYG1, mitochondrial**	C12orf10	GTHNG		
174	P21953	ODBB_HUMAN	**2-oxoisovalerate dehydrogenase subu...**	BCKDHB	VAHFT		
175	Q9UI32	GLSL_HUMAN	**Glutaminase liver isoform, mitochon...**	GLS2	GSHCG		
176	Q7Z4T9	CFA91_HUMAN	**Cilia- and flagella-associated prot...**	CFAP91	MSHAV		
177	Q92667	AKAP1_HUMAN	**A-kinase anchor protein 1, mitochon...**	AKAP1	KGHVS		
178	Q9NSE4	SYIM_HUMAN	**Isoleucine--tRNA ligase, mitochondr...**	IARS2	SNHQP		
179	P0DJ93	SIM13_HUMAN	**Small integral membrane protein 13**	SMIM13	MWHSV		Location not defined unequivocally
180	Q9HBX3	SNIT1_HUMAN	**Uncharacterized protein encoded by ...**	SND1-IT1	MSHHP		
181	Q8N6N6	NATD1_HUMAN	**Protein NATD1**	NATD1	MAHSA		
182	Q8N8F7	LSME1_HUMAN	**Leucine-rich single-pass membrane p...**	LSMEM1	MTHSS		
183	P0C5K7	CT62_HUMAN	**Cancer/testis antigen 62**	CT62	MMHTT		
184	Q96FZ5	CKLF7_HUMAN	**CKLF-like MARVEL transmembrane do...**	CMTM7	MSHGA		
185	Q96M85	YV008_HUMAN	**Putative uncharacterized protein FL...**		MSHSR		
186	C9J302	CD051_HUMAN	**Uncharacterized protein C4orf51**	C4orf51	MSHYF		
187	Q8NCL8	TM116_HUMAN	**Transmembrane protein 116**	TMEM116	MKHTQ		

188	Q9NPI7	KRCC1_HUMAN	**Lysine-rich coiled-coil protein 1**	KRCC1	MKHSK		
189	P62955	CCG7_HUMAN	**Voltage-dependent calcium channel g...**	CACNG7	MSHCS		
190	Q5T8R8	CI066_HUMAN	**Uncharacterized protein C9orf66**	C9orf66	MRHSV		
191	Q9UHP6	RSP14_HUMAN	**Radial spoke head 14 homolog**	RSPH14	MAHSQ		
192	Q7Z429	LFG1_HUMAN	**Protein lifeguard 1**	GRINA	MSHEK		
193	P26992	CNTFR_HUMAN	**Ciliary neurotrophic factor recepto...**	CNTFR	QRHSP		
194	Q8NFZ6	VN1R2_HUMAN	**Vomeronasal type-1 receptor 2**	VN1R2	MTHTL		
195	Q6ZTU2	E400N_HUMAN	**Putative EP400-like protein**	EP400P1	MQHVS		
196	Q69YU3	AN34A_HUMAN	**Ankyrin repeat domain-containing pr...**	ANKRD34A	MLHTE		
197	Q96A19	C102A_HUMAN	**Coiled-coil domain-containing prote...**	CCDC102A	MSHGP		
198	Q4AC99	1A1L2_HUMAN	**Probable inactive 1-aminocyclopropa...**	ACCSL	MSHRS		
199	Q9NXS3	KLH28_HUMAN	**Kelch-like protein 28**	KLHL28	MDHTS		
200	A6PVS8	LRIQ3_HUMAN	**Leucine-rich repeat and IQ domain-c...**	LRRIQ3	MFHGT		
201	Q96KN7	RPGR1_HUMAN	**X-linked retinitis pigmentosa GTPas...**	RPGRIP1	MSHLV		
202	Q5RHP9	ERIC3_HUMAN	**Glutamate-rich protein 3**	ERICH3	MSHSH		
203	Q6ZVL6	K154L_HUMAN	**UPF0606 protein KIAA1549L**	KIAA1549L	MDHTA		
204	Q13519	PNOC_HUMAN	**Prepronociceptin**	PNOC	TLHQN	propeptide	Secreted
205	P13232	IL7_HUMAN	**Interleukin-7**	IL7	MFHVS	signal peptide	
206	O14944	EREG_HUMAN	**Proepiregulin**	EREG	TVHQP	propeptide	Extracellular side of cell membrane
207	P04234	CD3D_HUMAN	**T-cell surface glycoprotein CD3 del...**	CD3D	MEHST	signal peptide	
208	P33681	CD80_HUMAN	**T-lymphocyte activation antigen CD8...**	CD80	MGHTR	signal peptide	
209	P21754	ZP3_HUMAN	**Zona pellucida sperm-binding protei...**	ZP3	RRHVT	propeptide	
210	P13688	CEAM1_HUMAN	**Carcinoembryonic antigen-related ce...**	CEACAM1	MGHLS	signal peptide	
211	Q7L985	LIGO2_HUMAN	**Leucine-rich repeat and immunoglobu...**	LINGO2	MLHTA	signal peptide	
212	Q13634	CAD18_HUMAN	**Cadherin-18**	CDH18	TAHHS	propeptide	
213	Q8N271	PROM2_HUMAN	**Prominin-2**	PROM2	MKHTL	signal peptide	
214	Q99665	I12R2_HUMAN	**Interleukin-12 receptor subunit bet...**	IL12RB2	MAHTF	signal peptide	
215	P39086	GRIK1_HUMAN	**Glutamate receptor ionotropic, kain...**	GRIK1	MEHGT	signal peptide	
216	O94772	LY6H_HUMAN	**Lymphocyte antigen 6H**	LY6H	AGHSP	propeptide	
217	O14798	TR10C_HUMAN	**Tumor necrosis factor receptor supe...**	TNFRSF10C	SSHYL	propeptide	
218	P14091	CATE_HUMAN	**Cathepsin E**	CTSE	SLHRV	activation peptide	Endoplasmic reticulum / Golgi apparatus
219	P05546	HEP2_HUMAN	**Heparin cofactor 2**	SERPIND1	MKHSL	signal peptide	

220	P56817	BACE1_HUMAN	**Beta-secretase 1**	BACE1	TQHGI	propeptide	
221	Q9Y3D3	RT16_HUMAN	**28S ribosomal protein S16, mitochon...**	MRPS16	MVHLT	transit peptide	Mitochondrion
222	Q9UK39	NOCT_HUMAN	**Nocturnin**	NOCT	MFHSP	transit peptide	
223	P15514	AREG_HUMAN	**Amphiregulin**	AREG	SGHYA	propeptide	Location not defined unequivocally

[a] The list was prepared on the basis of the analysis published in *Chem. Biodiversity* **2021**, *18*, e2100043.

[b] The list shows N-terminal sequences of human proteins i) without initiator methionine removal, ii) after initiator methionine removal, iii) after propeptide/signal peptide removal, and iv) propeptides/signal peptides cleaved from proteins.

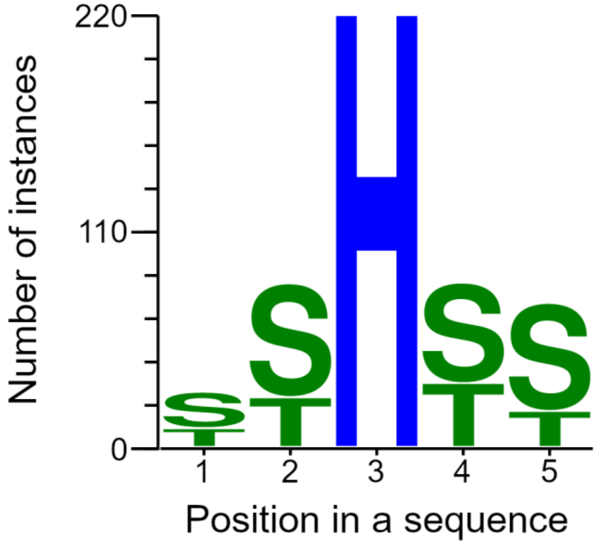

Fig. S7. Sequence logo of human proteins' ATCUN motif with serine or threonine residues, prepared using all sequences from Table S1. Only serine and threonine residues are shown for clarity. The height of every letter corresponds with the frequency of the respective amino acid residue in the given position. The logo was generated by WebLogo 3.7.4 (*Genome Res.* **2004**, *14*, 1188-1190; *Nucleic Acid Res.* **1990**, *18*, 6097-6100; http://weblogo.threeplusone.com).
